# Editorial: Regulatory Peptides in Neuroscience and Endocrinology: A New Era Begins

**DOI:** 10.3389/fendo.2019.00793

**Published:** 2019-12-04

**Authors:** Limei Zhang, David Vaudry, Colin H. Brown, Lee E. Eiden

**Affiliations:** ^1^Department of Physiology, Universidad Nacional Autonoma de México, Mexico City, Mexico; ^2^Normandie Univ, UNIROUEN, Inserm, Laboratory of Neuronal and Neuroendocrine Communication and Differentiation, Neuropeptides, Neuronal Death and Cell Plasticity Team, Rouen, France; ^3^Department of Physiology, University of Otago, Dunedin, New Zealand; ^4^National Institute of Mental Health, Intramural Research Program, NIH, Bethesda, MD, United States

**Keywords:** regulatory peptides, neuropeptide, incretin, gut-brain axes, translation

The Research Topic “Regulatory Peptides in Neuroscience and Endocrinology: A New Era Begins” represents the second such Research Topic gathered under the auspices of the International Regulatory Peptide Society, an international federation of scientists committed to progress in basic research on the biology and physiology of peptides, and translation of that basic research into clinical gains and public health benefit. The first summarized key contributions from RegPep2016 (1), held in Rouen, France July 13–17, 2016. This second corresponds to RegPep2018, held in Acapulco Diamante September 22–25, 2018, with the stated mission of emphasizing the emergence of peptides as therapeutic agents from the basic research on newly discovered regulatory peptides, and new roles of established regulatory peptides, *in vivo*.

We refer here to a new era in regulatory peptide research for two reasons. The first concerns the changing definition of “translation.” The concept of research translation as first formulated in the early twenty-first century, emphasized it as a separate discipline bridging basic and clinical research to accelerate public health gains. This included establishment of separate departments of translational science or translational medicine, to act as the “midwives” for rapidly carrying basic knowledge to the bedside of the patient. However, this formulation has risked the further “siloing” of scientific and clinical endeavor which the very concept of translational research/science was meant to overcome. We now tend to view translational research as simply all of the research that practitioners do, but performed with a mindfulness of therapeutic application. This is achieved by conferences such as RegPep, at which the actual progress made toward therapeutic fulfillment of promising leads is shared, monitored and assessed. Hence, this editorial overview.

Are we learning both positive and negative lessons about what really works, in the lab and the clinic, to advance understanding of how regulatory peptides perform their roles in systems physiology? Here, several major questions are emerging. These include: (i) how redundant are the functions of multiple peptides that converge on the same circuit nodes in the endocrine and nervous systems? Insulin secretion, for example, is controlled by incretins, neurotransmitters, hormones, paracrine factors, and metabolites-including the focal regulatory point itself, namely glucose. Yet, none of these is “redundant” in the sense of being merely duplicative. Rather, fine regulation of insulin secretion apparently requires all, and therefore therapeutic application of any one target of “insulin regulation” must be seen in the context of this integrative regulation, lest one step forward result in two back, due to counter-regulatory mechanisms intersecting with drug presentation. Regulatory peptides in the central nervous system also regulate glucose indirectly via control of food intake, by a mix of allostatic and homeostatic (or “reward” and “metabolic”) drives. The wish to exploit this complexity rather than be overwhelmed by it, is exemplified by the latest batch of incretin-based peptide therapies for management of diabetes (2). So, the new era beginning is one in which it is acknowledged that various treatments will represent opportunities unique to the variations among human physiologies, diets, motivations, threats, and habitual behaviors, and that animal models relevant to human disease will require a subtlety matching these variations.

*The presentations from RegPep2018 that have been contributed and selected here represent several distinct themes relevant to the new era of regulatory peptide research*.

The systematic study of sex differences is an explosive growth area in “personalized medicine:” sex is the most obvious of the personal differences to be considered in tailoring of therapy in psychiatric, metabolic, oncologic, and immunologic disease arenas to the individual. Male/female differences are not confined to the gonads and their neuroendocrine regulation, but also to the mechanisms whereby appetitive and aversive drives are prioritized, reflecting the behavioral choices of individuals. This intersection of metabolic and hormonal sexual dimorphism penetrant to behavioral dimorphism is illustrated by the contributions of Jaimes-Hoy et al. and Parra-Montes De Oca et al., which represent the first two contributions of this Research Topic. Focusing on the hypothalamic-pituitary-thyroid axis, these laboratories studied two stressors, maternal separation, and restraint, and their effects on the metabolic outcomes of administration of a high-fat diet and voluntary exercise on HPT axis activation, and on the “final outcomes” of body weight and fat content. Clearly, elucidating chains of causality across multiple intersecting endocrine regulatory loops is problematic. However, these contributors emphasize the overall finding that the response of the HPT axis to stress, and stress/metabolism/feeding behavior interactions, shows sexually dimorphic patterns. Fitting this information to human metabolism, feeding behavior, and health outcomes to illuminate gender-specific patterns, both learned and hard-wired, is now an obligatory task for neuroendocrinologists.

The mapping of peptidergic brain circuits, particularly in the mouse in which genetic manipulations and virally-mediated tract tracing can be accomplished, is an area of regulatory peptide research that has been progressing at a rapid rate for the past decade, aided in large part by the Allen Brain Project (www.brain-map.org/). Zhang et al. present here the whole-brain mapping of the afferent inputs to cortical corticotropin-releasing hormone (CRH) neurons. The critically important GABAergic interneurons of the cerebral cortex are increasingly seen as coordinated but functionally independent arrays, and one of the means of distinguishing them is via the neuropeptides that they mutually exclusively express. Neuropeptide expression can provide a marker that in addition allows promoter-specific genetic manipulation of these subpopulations, and ultimately a way of linking neuron structure and neurochemistry to brain circuit function. In this particular case, rabies virus was injected into cerebral cortex in a recipient mouse with the correct trans-genetic trappings to produce virions only in host neurons that are CRHergic (harbor a CRH-promoter-specific Cre element), those virions having the property of uptake and fluorescent marking of afferent cells (i.e., a retrogradely-labeling *in vivo*-generated “marker virion”). The plethora of inputs (to anterior cingulate cortical CRHergic interneurons) from cortex, thalamus, olfactory area, amygdala, basal forebrain, striatum, hippocampus, midbrain (e.g., ventral tegmental area), and hindbrain (e.g., raphe nuclei) raise several interesting questions, among them: (i) is the functional importance of such inputs proportional to their relative abundance (they range from <1% to 10–15%), (ii) is there any latent topology revealed by such studies?, (iii) if and when will such studies converge and combine to offer novel physiological insight into how CRH and other regulatory peptides function in different types of neurons, and can this information be parlayed into a “combinatorial signaling code” that can be exploited for therapeutical purposes in specific cerebral pathologies? The contribution of Dedic et al. suggests that there are specific neuropathology-related roles played by this very cerebral interneuronal population in the mouse. Thus, deleting CRH from these neurons, while sparing other CRHergic subpopulations throughout the brain, appears to promote stress resilience by damping stress-induced neuronal activation. Put another way, CRH function in this neuronal population seems to be to promote or exaggerate the importance of stress in altering brain function: in animals with CRH deficiency in these neurons but not in hypothalamus and other brain areas, CRH-related endocrine functions appear to be normal, while stress-triggered behaviors (anxiogenesis, for example) are blunted. This is very much in tune with results in other peptide systems, as in dissociation of endocrine and behavioral effects of PACAP in stress responding (3) and a potential harbinger of how sub-specialization of regulatory peptide synaptic function might be exploited therapeutically, should differential delivery to different brain regions (4), or biased signaling agonism and antagonism, be achievable in these functionally distinct systems.

A further intriguing example of discrimination between dynamic homeostatic states afforded by a brain peptidergic system is provided by the report of Head et al. for oxytocin (OT) effects on food intake at various stages of food intake, i.e., before vs. during food intake. A novel hunger discrimination protocol was employed to allow rats to distinguish, based on a two-lever operant procedure, between 2- and 22-h food deprivation, and the effect of i.p. OT. at a dose that diminishes food intake, on discrimination-driven response to these two “hunger states.” The authors conclude that OT affects neuronal activation (c-fos expression) in a broader network of neurons in the sated compared to the hungry state, and thus, with other supporting data including dynamic changes in OTR expression in brain, that OT likely has anorexigenic effects on continued feeding rather than on initiation of feeding when hungry. Given the complex interactions among peptide systems in hypothalamus that control both hunger and satiety, the notion that multiple phases of feeding (each in turn influenced by affect/mood, and other drives including threat aversion and thirst) can be modulated by peptidergic systems encourages further experiments that calibrate peptide effects on feeding to state and trait variables relevant to food intake. A more robust understanding of the motivation for feeding may mean that combinatorial approaches to multi-peptide control of feeding are on the horizon (Head et al.).

Vasopressin, like the closely-related oxytocin, has effects mediated both in the telencephalon, through synaptic actions via projections from hypothalamus, and in brain stem, perhaps via a combination of hormonal and neuronal peptide action at receptors there. Two contributions (LZ and LE are co-authors of the first report, and LZ is co-author of the second) by Hernández-Pérez et al., and Campos-Lira et al., feature the elucidation of vasopressinergic projections to the locus coeruleus, which are likely to gate stress- and attention-related motivation for various appetitive and aversive behaviors. Similarly to the OT system (see Head et al. above), vasopressin receptor expression seems to be an important dynamic regulator of how important peptide influence on allostatic behavioral responses will be for a given state-sexual, hormonal, metabolic, hydromineral-that the animal finds itself in upon exposure to stressors and other environmental contingencies (and see the very interesting contribution of Zetter et al. re: vasopressin's ectopic production and role in acceleration of infection in tuberculosis, suggesting vasopressin antagonism as a target in TB).

An increasingly important aspect of regulatory peptide action is brought to the fore in considering vasopressin's actions in the brain: the role of classical transmitter co-transmission in the actions of neuropeptides and other regulatory peptides. This is of fundamental importance both centrally and peripherally. The Hernandez-Perez report (Hernández-Pérez et al.) (two authors of this commentary are co-authors of this report) reveals that the vasopressinergic projections to the locus coeruleus, like those to other brain areas including hippocampus, amygdala, and lateral habenula, are also glutamatergic. As pointed out by Hokfelt and colleagues some years ago (5), release of classical transmitters from small synaptic vesicles is likely to be a roughly linear function of neuronal firing frequency, whereas release of regulatory peptides from large dense-core vesicles occurs largely only at high firing frequencies, in part because a higher concentration of intraterminal calcium is required for LCDV vs. SSV release. Although peptides are preferentially released at high firing frequencies, however, this is also the point at which classical transmitter release is also maximal. Thus, attention to the role(s) of small transmitter-regulatory peptide secreted/released *together* must be a part of the analysis of how regulatory peptides act in both the central and peripheral nervous systems (though not for their actions as hormones).

The contribution of Lindberg et al. (LE is a co-author of this report) to the Research Topic emphasizes this latter point. It has previously been shown that the phase-advancing effects of light during late night in the mouse *in vivo*, but not the phase-delaying effects of light during early night, is PACAP-dependent (does not occur in PACAP-deficient mice). In slices of hypothalamus containing suprachiasmatic nucleus (SCN) *ex vivo*, glutamate substitutes for light in driving both late-night phase advance and early-night phase delay, and, as *in vivo*, the former does not occur in slices from PACAP-deficient mice, or from enucleated mice in which denervation of the retinohypothalamic tract input to the SCN has occurred. The permissive role of PACAP in glutamate-dependent phase advance in the circadian pacemaker center of the brain, the suprachiasmatic nucleus, in response to PACAP/glutamate co-release from retinohypothalamic projections from intrinsically photosensitive retinal ganglion cells (ipRGCs) of the retina, correlates with waxing and waning of PACAP mRNA expression in the RGC layer of the retina *in vivo*.

The contribution on kisspeptin expression and function in the seahorse hypothalamus illustrates a critical mainstay of regulatory peptide research: comparative physiology and neuroendocrinology. Since the very inception of the field, comparisons between mammalian and non-mammalian systems have informed the understanding of regulatory peptides due to conservation of critical structural elements and conservation of critical homeostatic functions among diverse species. The report by Zhang et al. reveals that interaction between kisspeptin and GnRH neurons are critical for sexual maturation initiated in the hypothalamus in response to both brain-intrinsic and brain-extrinsic cues, and that these interacting systems have been honed by evolution not only for sexually segregated reproductive function as found in mammals, but for more variegated sexual options in other species. Knowledge of reproduction of all extant species enriches our understanding of the planetary lifeweb, with practical implications in this case for fish husbandry, as well as the co-evolution of ligand-receptor dyads, and dyadic interactions, that extends beyond these particular brain regulatory peptides (6–8).

Galanin is an intriguing peptide first discovered by chemical means as an amidated gut peptide, rather than based on a search for the structure of a known physiotropic “factor.” Following its structural elucidation, galanin was rapidly shown to be synthesized not only in gut but in brain, adrenal medulla, pituitary and peripheral nervous system. Galanin is truly a “jack of all trades,” even among regulatory peptides (9), and the contribution of Yun et al. exemplifies this. True to the theme of peptide/receptor duplication and redundancy, with evolutionarily acquisition of specialization for divergent ligand-receptor dyads, the novel neuropeptide spexin interacts with galanin receptors types 2 and 3. The structure of spexin, in comparison with that of galanin, has allowed spexin-based drug development with specificity for the latter receptors. In this case, the GALR-2-specific agonist CG2A, when administered intraventricularly in CORTI mice, led to recovery of body weight in these mice (but to weight loss in normal mice). Fear memory consolidation was attenuated, while extinction of fear memory was accelerated, in SG2A-treated mice. These results suggest multiple actions of CG2A along the neural axis, and interestingly, intranasal administration of CG2A showed a similar profile with respect to memory and feeding, suggesting that the compound penetrates the blood-brain-barrier, or at least reaches its key sites of action via specialized transport to those brain regions. These experiments may well be the beginning of further interest in spexin-related peptides as treatments for mood and metabolic disorders, which have a high degree of co-morbidity.

The last four contributions to this Research Topic are reviews rather than original reports, and offer an expanded view of past progress and anticipated future directions for the regulatory peptide field.

Corbière et al. review (DV is a co-author of this report) the cohort of biochemical, bioinformatic, genetic, and pharmacological tools available for combing the genomes, transcriptomes, proteomes, and peptidomes of diverse species and tissues for novel peptides in vertebrates with bona fide biological activity: the case of nociception is used as a primary illustration. The strategies for identifying bioactive neuropeptides depend largely upon the “game plan” of the investigating teams. Goals can be oriented toward discovery, systems biology, or a combination of both. A clear algorithm for identifying (novel) bioactive neuropeptides is not yet in view, likely because diversity of biology begets diversity of technology.

The neuroanatomical superficiality of the review of Gorky and Schwaber is, in a way, refreshing after decades of unnecessary obfuscation about whether to call intrinsic cholinergic gut innervation “parasympathetic” or “enteric.” Intrinsic cholinergic neurons of the gut are, in fact, parasympathetic, and in that sense the intrinsic cholinergic component of the enteric nervous system is in fact not magically different from intrinsic cholinergic parasympathetic innervation of heart, kidney, lung, and other visceral organs. What does make the gut unique, with respect to its parasympathetic innervation, is the plethora of peptide-elaborating cells populating the gastrointestinal tract that are contacted by parasympathetic post-ganglionic (intrinsic) cholinergic neurons, and release paracrine, incretin, autocrine, and hormonal factors (the authors emphasize CCK, VIP, somatostatin and GLP-1 but there are many others). While the “conceptualization of a parasympathetic endocrine system” is in some ways incompletely thought-out in this contribution, the idea of it is a useful heuristic for re-evaluation of many decades of classical physiological observations of the effects of vagotomy on gastrointestinal function, and of the signaling of the gut to the brain, with the aid of modern imaging, tracing, genetic, and biochemical tools. We recommend the excellent review of Powley et al. (10) as a companion to the one offered here in this collection of reports and reviews.

This Research Topic closes with two contributions on polypeptides that might at first blush not seem to fit neatly into the category of “regulatory peptides” yet illustrate several facets of current endeavor in this rich field. The report by Loh and colleagues reviews current information about a new role for carboxypeptidase E (CPE) in neuroprotection. The importance of CPE as a processing enzyme in the chain of intracellular transformations leading from prohormone to peptide hormone has been long appreciated in regulatory peptide biology. CPE has a second role, however, wholly independent of its prohormone processing function, which is to act as a bioactive secreted peptide in its own right, mediating rather dramatic effects on neuronal protection against stress, and primarily in the hippocampus, one of the brain centers for processing of associated sensory signals in a way that allows basis of future behavior on past experience. It is fitting that the report of Lovejoy et al. is the final contribution of this collection, both original and synthetic, on the state-of-the-art of regulatory peptide research, and its translational potential. Teneurins are polypeptide ligands for receptors within the adhesion GPCR family. The ligand-receptor dyad mediates various facets of intracellular communication that must occur in synaptogenesis and other neurodevelopmental events. A peptide cleaved from the extracellular teneurin domain, called TCAP (teneurin C-terminal associated peptide) bears structural similarity to several family B peptides (including CRH, calcitonin, and secretin), and its receptor (latrophilin) is structurally related to the B family GPCRs. Whether or not TCAPs represent a primordial family B functional equivalent, TCAP-derived peptides may represent both tool pharmacological agents, and potential therapeutical avenues, for modifying family B-related neuroendocrine function and stress-related affective behaviors and disorders. TCAP pharmacology and, ultimately therapeutics, may reflect evolutionary either serendipity or the harvesting of evolutionary bricolage. In either case, regulatory peptide research is the richer for embracing both.

We urge the reader to peruse the contributions of this Research Topic as examples of this emerging new landscape, in which all aspects of regulatory peptide research are viewed in the largest possible physiological, clinical, and therapeutic perspective, so that all new experimental information, whether designed, of nature, or created from careful medical observation, is thoughtfully integrated.

## Author Contributions

All authors contributed to the writing and editing of this introduction.

### Conflict of Interest

The authors declare that the research was conducted in the absence of any commercial or financial relationships that could be construed as a potential conflict of interest.
